# Surgical Management of a Splenic Abscess: Case Report, Management, and Review of Literature

**DOI:** 10.7759/cureus.28567

**Published:** 2022-08-30

**Authors:** Courtney L Evans, Molly K Triggs, Jaydip Desai, Juan Campos

**Affiliations:** 1 Department of Research, Alabama College of Osteopathic Medicine, Dothan, USA; 2 General Surgery, Coosa Valley Hospital, Sylacauga, USA

**Keywords:** escherichia coli, laparoscopic appendectomy, splenic abscess, infectious disease, general surgery

## Abstract

Splenic abscess is a rare infection that may develop from a multitude of causes. There are several different microorganisms implicated in pathological formation including Staphylococci, Streptococci, Salmonella, and Escherichia coli. Antibiotics are the first line of therapy in treatment with eventual surgical intervention. It is imperative to have surgical intervention performed due to increased rates in mortality with only medical management. However, specific treatment guidelines in the management of splenic abscess have been unclear due to the low number of documented cases. We report the case of a splenic abscess in thirty-year-old female two months following an appendectomy. The goal of this case report is to help provide additional context into management and treatment options for splenic abscess using literature review.

## Introduction

Splenic abscesses are uncommon infections with a reported incidence of 0.05-0.7% [1]. One of the leading causes in the development of splenic abscess is seen in patients with a history of bacteremia. The proposed mechanism occurs secondary to hematogenous bacterial seeding, where microorganisms travel and spread from other contiguous sites of infection such as endocarditis [2]. Apart from primary infections, they may also occur during embolization procedures such as splenic artery pseudoaneurysm coiling [3]. Other notable risk factors include immunosuppression, diabetes, cardiac disease, neoplasia, or inciting traumatic events [1,3]. There are many causative organisms implicated in the pathologic development of splenic abscesses. The more common microorganisms isolated from abscess cultures include Staphylococci, Streptococci, Salmonella, and Escherichia coli [1]. However, there have been rare instances where splenic abscesses of fungal origin were diagnosed in immunocompromised patients. For example, Phyu et al. documented one case of splenic abscess as a rare presentation of Blastomycosis [4]. Furthermore, tuberculosis and malaria have been implicated as causative agents in developing countries. Splenic abscess, therefore, can be caused by a wide variety of microbial pathogens.

The diagnosis of a splenic abscess is often diagnosed through clinical suspicion. The triad of fever, leukocytosis, and left upper quadrant pain are only present approximately one-third of the time in patients with a suspected splenic abscess [5]. Therefore, radiological findings are confirmatory in the diagnosis. Increased usage of ultrasonography (US) and computed tomography (CT) have allowed for improved detection in recent years [6]. Treatment first involves initiating broad-spectrum antibiotics. Different surgical modalities can then be offered based on several factors. While the gold-standard treatment has been debated to be splenectomy, there have been more recent usage of percutaneous guided aspiration using CT guidance as a bridge in conjunction with antibiotics [6]. Compared to splenectomy, intervention with percutaneous drainage has not shown a difference in mortality between treatment and control groups [6]. However, if left untreated or treated only with antibiotics, mortality rates can range anywhere from 60-100% [7]. The natural history and treatment guidelines of splenic abscess are not well documented in literature due to the low incidence described earlier. We present a rare case of a diagnosed splenic abscess caused by Escherichia coli in a patient with recent appendicitis. Our case report aims to increase awareness for prompt diagnosis with the need for surgical intervention in addition to standard medical therapy.

## Case presentation

We present the case of a 30-year-old female who had undergone a laparoscopic appendectomy two months prior for confirmed appendicitis. Her past medical history was significant for chronic kidney disease, diabetes mellitus, and hypertension. One month following the appendectomy, she was evaluated in the local emergency department due to reports of persistent abdominal pain. Radiological findings ordered during that visit included an MRI without contrast, which demonstrated a lobulated area with a high T2 signal within the spleen which extended from the superomedial aspect to the posterior mid-spleen. Initially, this was suspected to be a splenic infarct due to the rarity of a splenic abscess. She was discharged with antibiotics and advised to follow up as needed.

**Figure 1:**
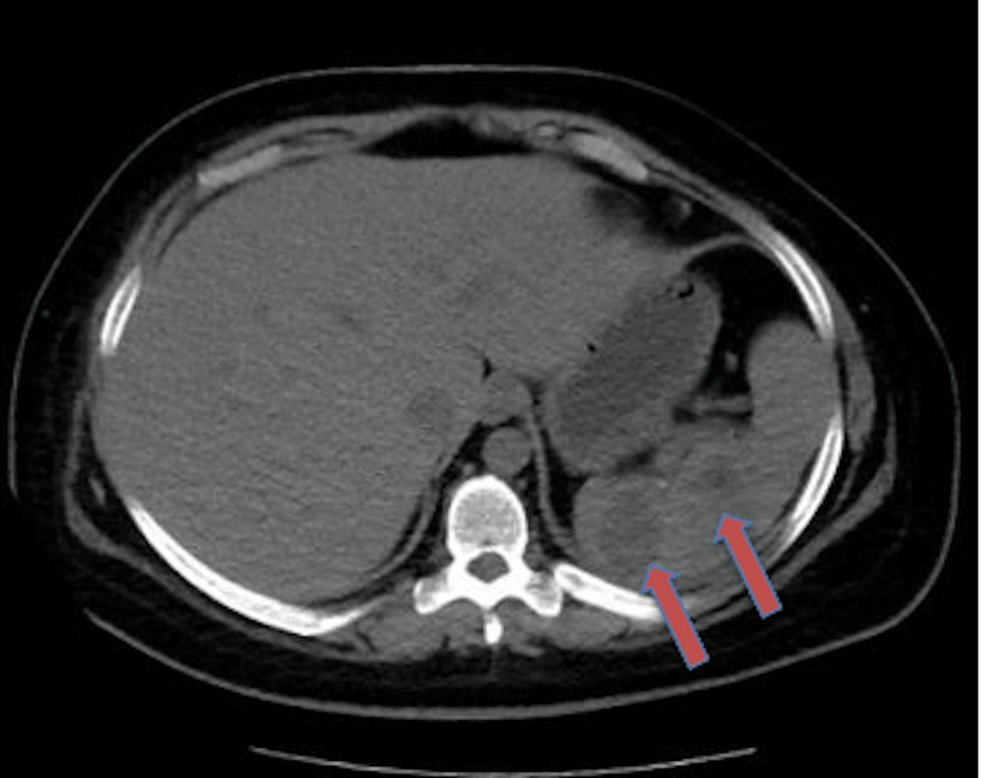
CT of the abdomen without contrast demonstrating hypodense areas of the spleen, consistent with abscess formation given the clinical presentation.

The patient presented again to the emergency room two months after her appendectomy with a chief complaint of left upper quadrant and chest wall pain for two days. CT abdomen/pelvis without contrast demonstrated progression of hypodensity in the spleen (Figure 1).

The study was limited due to the patient’s inability to tolerate IV contrast. It did not fit a classical radiologic picture of a splenic infarct; therefore other diagnoses were considered. Due to the clinical history and left upper quadrant pain, fever, and leukocytosis, the diagnosis of a splenic abscess was suspected. The patient was taken to the operating room after providing consent and underwent an open splenectomy. Laparoscopic intervention was not performed due to surgeon preference given the rural setting of the hospital it was performed at. During the operation, the entire spleen was found to be ischemic. Dissection of the spleen resulted in back bleeding from the upper spleen, and spillage of pus into the abdominal cavity was noted. Proper hemostasis was obtained, and cultures were procured. Cultures were positive for Escherichia coli and negative for anaerobic growth.

**Figure 2:**
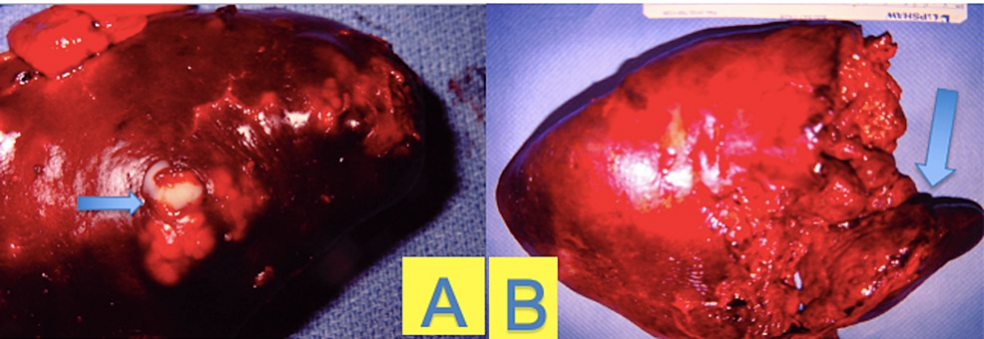
Gross pathology specimens of splenic abscess. Panel A shows pus formation within the spleen. Panel B shows parenchyma consistent with a dark red appearance indicating a late stage of inflammation. Reprinted from the University of Arizona at https://surgicalimages.arizona.edu with permission.

Pathology report demonstrated a spleen weighing 296 grams, measuring 12.5 x 8.5 x 6 cm. Peripherally on the diaphragmatic surface, yellow adipose tissue was approximately 5 x 2 cm in dimension. Adhered to that segment, was a light gray softened and necrotic surface tissue with a 5.5 x 2.5 cm dimension. Central to the peripheral hilar surface was a superficial decortication with 8 x 5 cm of red-brown tissue. Sectioning demonstrated softened and friable tissue beneath the area of light tan surface discoloration. The remaining parenchyma presented a solid and homogeneous dark pink-red appearance within the central portion beneath the focal disruption on the diaphragmatic surface. An example of splenic abscess is provided (Figure 2).

It was suspected that the abscess resulted from bacteremia secondary to appendicitis. The patient was seen for follow-up in the clinic two weeks later and recovered without postoperative complications from the surgery.

## Discussion

Splenic abscess is a fatal condition when left untreated. Prompt recognition using known risk factors is critical in diagnosis. It is uncommon for immunocompetent patients to develop a splenic abscess [8]. Therefore, the diagnosis may be challenging due to variability in clinical presentation. In the case of our patient, her only known risk factors included abdominal surgery without recent trauma and diabetes mellitus. Initial physical exam findings for most patients include fever and diffuse abdominal or left upper quadrant pain [4]. Additional clinical, radiological, and laboratory findings include splenomegaly, leukocytosis, and left-sided pleural effusions [3]. Our patient reported isolated abdominal and chest wall pain with no other symptoms. Furthermore, her radiological study initially was suggestive of a splenic infarct rather than an abscess. Abnormal gas content, subscapular extension, cystic lesions, extracapsular fluid collection, and progressive enlargement of lesions are more common findings of splenic abscess [4]. The use of CT imaging helped demonstrate hypodensity in her spleen, thereby establishing the proper diagnosis that led to eventual splenectomy with symptom resolution.

Due to the rarity of presentation, diagnosis and treatment guidelines have not been well established. Improper diagnosis of splenic abscess can result in a high mortality rate, extending beyond 70% [9]. However, this is reduced to less than 1% with accurate diagnosis and appropriate treatment. Treatment can include a myriad of options based on patient presentation and severity of the disease. Splenectomy was once considered the standard primary treatment when abscess formation develops. However, literature has suggested that patients may be treated with different, more conservative approaches [8]. The causative microorganism must be identified with a proper diagnosis. This is mainly done using either blood cultures or CT-enhanced percutaneous drainage. Furthermore, treatment may also depend on the size of the abscess. It has been suggested that treatment with intravenous antibiotics alone in splenic abscesses less than 4 cm in diameter. Additional treatment options for more extensive or multilocular abscesses include percutaneous drainage with ultrasound or computed tomography guidance or splenectomy [5]. Recent literature has shown percutaneous drainage to have a higher yield over blood cultures when confirming causative agents [10]. However, it is debated whether to recommend only using aspiration when multiple blood cultures report non-confirmatory findings given a fully established splenic abscess [9]. Percutaneous aspiration also has a documented failure rate between 14-70%, making it a less favorable option for some physicians [6]. Nevertheless, antibiotics are crucial to management irrespective of surgical intervention. Treatment of choice should be made using sensitivity assays after making a clear diagnosis using cultures or aspiration.

## Conclusions

Due to the infrequent presentation of splenic abscesses, there are no specific guidelines regarding the diagnosis and treatment, and the best mechanism of management is still undecided. More information is needed to enhance the interpersonal management of splenic abscesses and to strengthen the evidence-based care provided to patients.
